# Environmental effect of air versus gas tamponade in the management of rhegmatogenous retinal detachment VR surgery: A multicentre study of 3,239 patients

**DOI:** 10.1371/journal.pone.0263009

**Published:** 2022-01-26

**Authors:** George Moussa, Walter Andreatta, Soon Wai Ch’ng, Hadi Ziaei, Assad Jalil, Niall Patton, Tsveta Ivanova, Kim Son Lett, Dong Young Park

**Affiliations:** 1 Birmingham and Midland Eye Centre, Sandwell and West Birmingham Hospitals NHS Trust, Birmingham, United Kingdom; 2 Birmingham and Midland Eye Centre and Academic Unit of Ophthalmology, University of Birmingham, Birmingham, United Kingdom; 3 Manchester Manchester Royal Eye Hospital, Manchester University Hospitals NHS Foundation Trust, Manchester, United Kingdom; 4 Kantonsspital Winterthur, Winterthur, Switzerland; 5 University of Zurich, Zurich, Switzerland; 6 University Hospital Coventry and Warwickshire, University Hospitals Coventry and Warwickshire NHS Trust, Coventry, United Kingdom; Massachusetts Eye & Ear Infirmary, Harvard Medical School, UNITED STATES

## Abstract

**Purpose:**

To report the potential reduction of carbon emissions by utilising air-tamponade (AT), where possible, instead of fluorinated gases in the management of rhegmatogenous retinal detachment (RRD). We compared the carbon CO_2_ emissions produced at two large tertiary referral vitreoretinal (VR) centres where RRD are exclusively repaired using fluorinated gases to a tertiary VR mass of each gas used according to the Intergovernmental Panel on Climate Change.

**Materials and methods:**

Retrospective, continuous, comparative multicentre study of all procedures using fluorinated gases between 01/01/17-31/12/20 at the Manchester Royal Eye Hospital (MREH) and Birmingham and Midland Eye Centre (BMEC), and between 01/01/19-31/12/2020 at the University Hospitals Coventry and Warwickshire (UHCW).

**Results:**

We report on 3,239 (SF_6_:1,415 [43.7%], C_2_F_6_:1,235 [38.1%], C_3_F_8_:541 [16.7%], Air:48 [1.5%]) procedures. UHCW and BMEC utilise single use 30ml and 75ml cannisters, respectively and MREH use multi-use gas cylinders. UHCW used AT in 48 (70%) of RRD repairs. Mean equivalent mass CO_2_/patient was MREH:115.9kg, BMEC:7.9kg and UHCW:1.9kg. If assuming all centres used 30ml cannisters, the mean equivalent mass CO_2_/patient was MREH:3.5 kg, BMEC:3.1kg and UHCW:1.9kg. AT enabled UHCW to greatly reduce the need for the most environmentally damaging SF_6_ gas, leading to lower CO_2_ emissions by 47.0% and 41.1% compared to MREH and BMEC, respectively.

**Conclusion:**

We demonstrate how AT vs. the fluorinated gases can reduce in carbon footprint in the management of RRD. Further studies are required to determine the most ‘environment-friendly’ intraocular tamponade without compromising patient outcomes centre that also routinely employs AT in selected RRD cases.

## Introduction

The fluorinated gases used in vitreoretinal (VR) surgery which include sulphur hexafluoride (SF_6_), hexafluoroethane (C_2_F_6_) and octafluoropropane (C_3_F_8_) are among the most potent greenhouse gases [1]. The National Health Service (NHS) in the United Kingdom (UK) is the largest funded healthcare system in the world and it contributes to 5.4% of UK’s greenhouse gases’ production [2]. SF_6_ has been identified in the Kyoto Protocol as one of six gases which require strict regulation in order to reduce global warming [3].

In October 2020, the NHS committed to becoming ‘carbon net zero’ by 2040 [2]. There is increasing evidence in the literature on the excellent safety profile of air tamponade (AT) compared to gas in repairing selected rhegmatogenous retinal detachments (RRD) and some authors suggested utilising AT to reduce carbon emissions [4].

In this paper we primarily reported the potential reduction of carbon emissions by utilising AT instead of fluorinated gases in the management of RRDs. In our analysis we compared the CO_2_ emissions produced at two large tertiary referral vitreoretinal (VR) centres where RRD are exclusively repaired using fluorinated gases to a tertiary VR centre that also routinely employs AT in selected RRD cases.

## Materials and methods

This is a retrospective, continuous, comparative multicentre trial of all RRD repaired by pars plana vitrectomy (PPV). Three tertiary hospital centres were included in the study: Manchester Royal Eye Hospital (MREH), Birmingham and Midland Eye Centre (BMEC), and University Hospitals Coventry and Warwickshire (UHCW). For MREH and BMEC, we included all RRD surgeries over a period of four years, between the 1^st^ of January 2017 to the 31^st^ of December 2020. For UHCW, data were available for consecutive cases of a single surgeon (DYP) over a two-year period, from the 1^st^ of January 2019 to the 31^st^ of December 2020.

At UHCW, the inclusion criteria for operating with AT were based on those from the Pneumatic Retinopexy versus Vitrectomy for Retinal Detachment (PIVOT) trial: i) A single retinal break or a group of breaks, no larger than one clock hour in detached retina, ii) All breaks in detached retina to lie above the 8 and 4 o’clock meridian and iii) Breaks or lattice degeneration in attached retina at any location (even inferior) were allowed [5]. AT was avoided in the presence of proliferative vitreo-retinopathy (PVR) grade C [6] and these data were collected prospectively. At BMEC and MREH, the choice of gas tamponade was at the discretion of the surgeon.

### Data acquisition

For MREH, data were extracted from a centralised database of all VR procedures performed, recorded on Microsoft Access. For BMEC, data were acquired from the electronic patient records (EPR, Medisoft Ophthalmology, Medisoft Limited, Leeds, UK). Finally, UHCW data were taken from DYP’s surgical logbook.

### Environmental factor calculations

The environmental aspect of gas tamponade was performed by converting millilitre of gas to mass (g) using the modified ideal law gas formula at standard temperature and pressure (STP). Intraocular gas masses were then converted to their Global Warming Potential (GWP) over 100 years (GWP100). GWP is defined as the heat absorbed by greenhouse gases in the atmosphere, as a multiple of the equivalent heat that would be absorbed by the same mass of carbon dioxide (CO_2_) [1]. The GWP of CO_2_ is one. In addition, as the fluorinated gases persist in the atmosphere far longer than CO_2_, their GWP increases with time. The fluorinated gases will therefore have a lower GWP20 (GWP over 20 years) than the GWP100 and GWP500 as demonstrated in Table 1 [7]. This table was adapted from the Intergovernmental Panel on Climate Change (IPCC) second assessment report [8].

**Table 1:** Global Warming Potentials at different time periods.

Gas	Lifetime (years)	Global Warming Potential (Time Horizon)		
		20 years	100 years	500 years
**CO**_**2**_	Variable (100–300)	1	1	1
**SF**_**6**_	3,200	16,300	23,900	34,900
**C**_**2**_ **F**_**6**_	10,000	6,200	9,200	14,000
**C**_**3**_ **F**_**8**_	2,600	4,800	7,000	10,100

Table adapted from the second Intergovernmental Panel on Climate Change (IPCC) report.

In this study we used the most current GWP100 reference values of the IPCC fifth assessment report [9].

The VR units in our hospital centres utilise three different gas delivery systems: i) UHCW: 30ml single-use gas cannisters (Arcadophta, Toulouse, France); ii) BMEC: 75ml multi-use gas cannisters (ALCHIMIA Srl, Padova, Italy); iii) MREH: traditional gas cylinders (British Oxygen Company [BOC] Healthcare, UK). For MREH, a staff survey revealed that approximately 30-50ml of gas is extracted for each operation from the respective cylinders. However, to reflect real life efficiency gas cylinder use, the mean amount of gas used per operation was calculated by dividing the amount of gas in complete cylinders used (as per pharmacy order history) by the number of operations performed using that respective gas within the study period.

The three gas delivery systems employed in this study produce variable CO_2_ emissions when analysing the same type of fluorinated gas. The two main aspects that affect equivalent CO_2_ mass emissions in using fluorinated gases in VR surgery are i) the number of procedures performed with each gas, and ii) the gas delivery system used [1].

As the gas delivery system has a profound impact on the amount of fluorinated gas used per procedure [1], and this manuscript aims to assess the number of fluorinated gas procedures that could be reduced by using AT, the gas delivery system must be controlled for, to allow comparison between different centres in this study. To adjust for this confounding factor, we have adjusted it to the equivalent CO_2_ production by assuming that every gas delivery system had been employed at each centre. The calculated mass of equivalent CO_2_ was performed with a conversion tool by the United States Environmental Protection Agency [10].

### Surgical method

Surgical correction of RRD was performed by transconjunctival 23/25/27-gauge PPV, vitreous-base trim and satisfactory removal of sub-retinal-fluid (SRF). PFCL was used at discretion of the surgeon. Fluid-air exchange (FAX) was performed in all patients. Subsequent gas-air exchange was done in the fluorinated gas group. Retinopexy was performed with localised endo-laser and/or external cryotherapy. Sclerotomies were sutured with 8–0 vicryl if air or gas leak through the wound is suspected.

### Statistical analysis

Statistical significance was defined as p<0.05. Prior to analysis, normality of continuous variables was assessed using the Shapiro-Wilk test, and found not to be normally distributed. Hence, data are primarily reported as medians and interquartile ranges (IQRs) throughout. Mann Whitney U and Kruskal Wallis Test were used to compare two and three independent continuous variables, respectively. Fisher exact test and Chi-Squared test were used for nominal variables. Bonferroni correction was applied for multiple statistical analysis. All statistical analysis was performed using IBM SPSS Statistics for Windows, Version 27.0 (IBM Corp, Armonk NY).

### Ethical approval/consent to participation

This study was registered and approved by our local clinical effectiveness team (Clinical Effectiveness Department, Sandwell General Hospital: reference number: 1593). As this was a retrospective anonymized study, as per our local protocol from our Clinical Effectiveness Department, and as per national guidelines from the National Code of Clinical Research, and the Health Research Authority (HRA), this study has ethical approval exemption and no patient consent was required for participation [11,12]. All procedures were completed prior to the design of this study. Patients were diagnosed and treated according to local guidelines and agreements and written consent from patients was acquired prior to all procedures as clinically indicated. This study does not report on the use of new or experimental protocols.

## Results

A total of 3,239 RRD cases were analysed. Table 2 shows the type of intraocular tamponade used for RRD repair. Table 3 reveals the mass of gas used per operation and its CO_2_ equivalent, depending on the three different gas delivery systems available. As the variability of gas delivery system acts as a significant confounding factor in the GWP100 CO_2_ equivalence calculation we assumed that all operations were performed using 30ml single use gas cannisters across all the three VR units in one of our analysis of the data (Fig 2).

**Table 2:** Tamponade number by centre for rhegmatogenous retinal detachment repair.

	Total	MREH	BMEC	UHCW
Total	3,239	1,590	1,489	160
**Tamponade**				
Air	48 (1.5%)	**-**	**-**	48 (30.0%)
SF_6_	1,415 (43.7%)	880 (55.3%)	511 (34.3%)	24 (15.0%)
C_2_F_6_	1,235 (38.1%)	625 (39.3%)	530 (35.6%)	80 (50.0%)
C_3_F_8_	541 (16.7%)	85 (5.3%)	448 (30.1%)	8 (5.0%)
Primary RRD	2,898 (89.5%)	1,425 (89.6%)	1,328 (89.2%)	145 (90.6%)
Macula on*	1,560 (49.8%)	739 (46.6%)	762 (54.9%)	59 (37.8%)

*macula status could be determined in 109 patients (3.4%).

**Table 3:** Fluorinated gas mass per operation by source.

		30ml Cannister	75ml Cannister	Actual Cylinder usage	Theoretical 25ml per operation Cylinder
SF_6_	Mass per unit (g)	0.195	0.489	6.633	0.163
	CO_2_ equivalent (g)	4,594.1	11,485.3	155,887.2	3,828.4
C_2_F_6_	Mass per unit (g)	0.185	0.462	3.650	0.154
	CO_2_ equivalent (g)	2,050.4	5,126.0	40,510.9	1,708.7
C_3_F_8_	Mass per unit (g)	0.252	0.629	23.121	0.262
	CO_2_ equivalent (g)	2,239.7	5,599.3	205,780.3	1,866.4

Based on GWP100 values from the Intergovernmental Panel on Climate Change (IPCC) fifth assessment report (2014).

**Fig 1:**
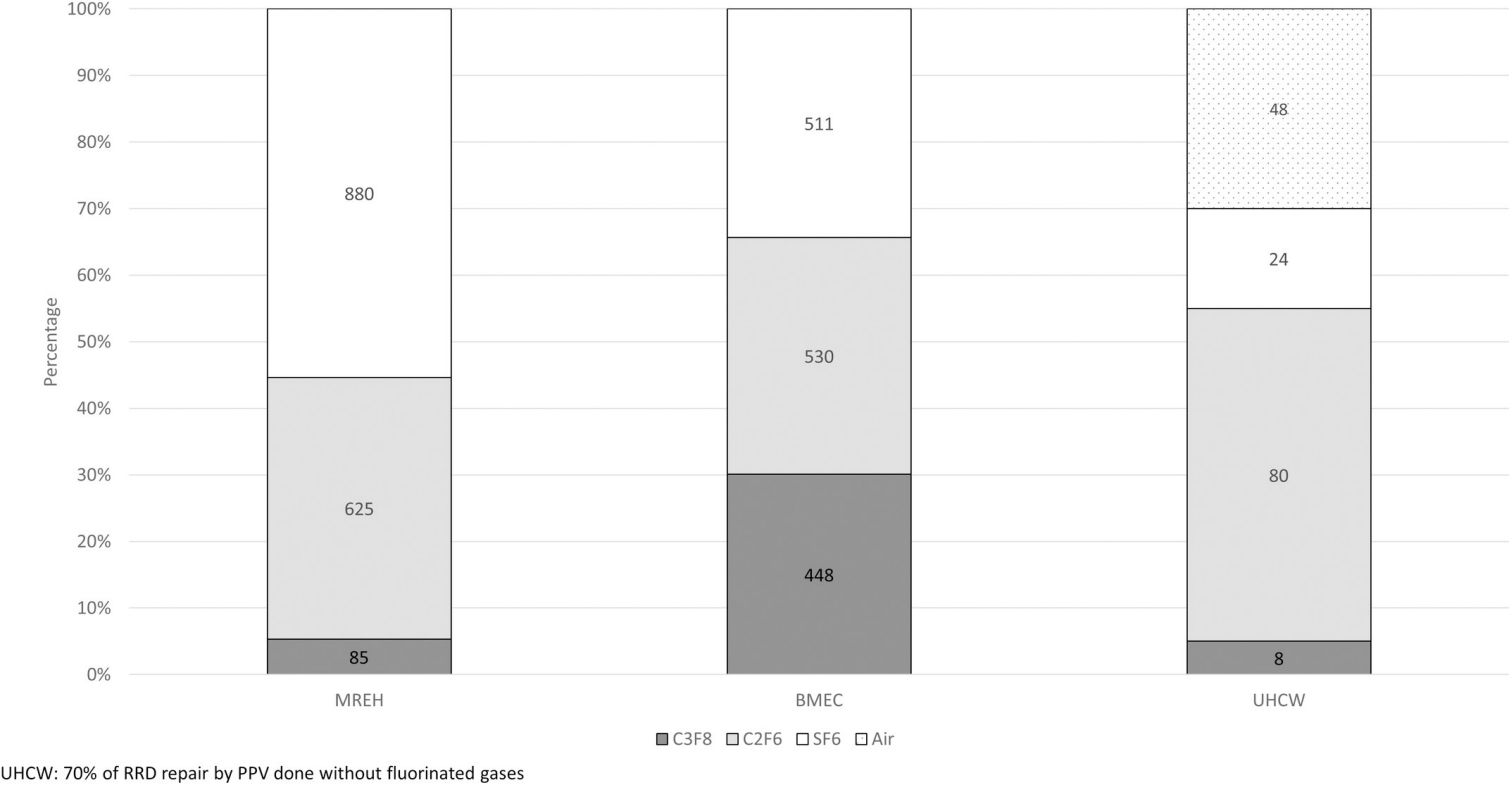
Proportion of air and gas tamponade used for RRD by centre. UHCW: 70% of RRD repair by PPV done without fluorinated gases.

**Fig 2:**
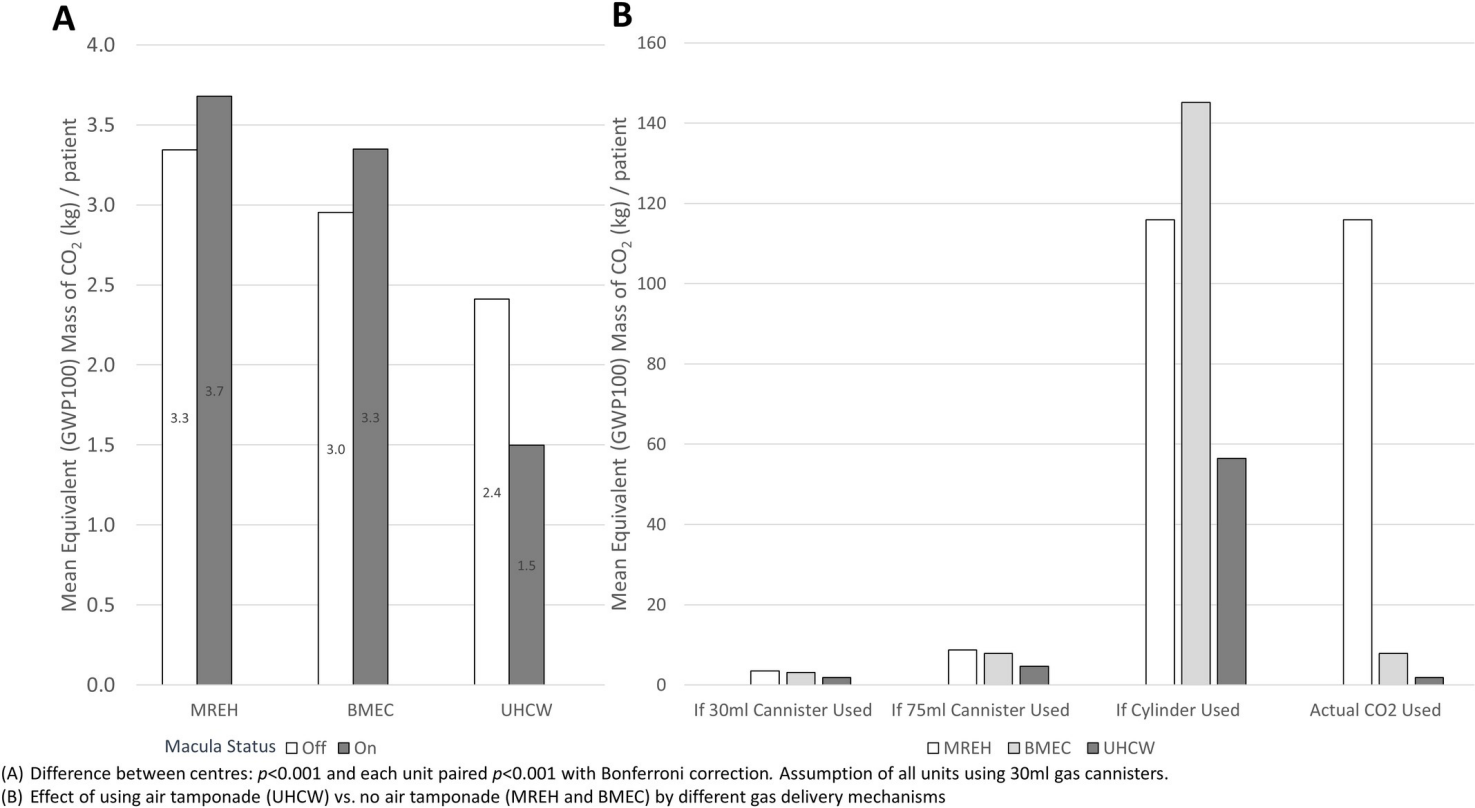
Mean equivalent (GWP100) mass of CO_2_ (kg) / patient for RRD repair. (A) Difference between centres: *p*<0.001 and each unit paired *p*<0.001 with Bonferroni correction. Assumption of all units using 30ml gas cannisters. (B) Effect of using air tamponade (UHCW) vs. no air tamponade (MREH and BMEC) by different gas delivery mechanisms.

Table 2 and Fig 1 demonstrate that at UHCW, 30% of all RRDs were repaired using AT which led to zero carbon emissions due to tamponade gas choice. Fig 2A shows the mean CO_2_ mass equivalence if all centres had used the most environmentally friendly delivery system (the 30ml cannisters) as fluorinated gas source. In this scenario, MREH showed the highest proportion of SF_6_ use and the greater carbon footprint while UHCW reported the lowest CO_2_ emission per patient. The use of AT enabled UHCW to greatly reduce the need for the most environmentally damaging SF_6_ gas, leading to lower CO_2_ emissions by 47.0% and 41.1% compared to MREH and BMEC, respectively. However, BMEC also showed a 10.0% reduction in carbon emissions per patient compared to MREH due to different proportion of type of gas tamponade used (p<0.001 in all paired comparisons between centres with Bonferroni correction).

Fig 2B clearly illustrates the impact of AT on reducing greenhouse emissions in RRD repair with the gas delivery system used. Therefore, the absolute reduction in greenhouse emissions will be more significant as the carbon footprint of the gas source increases. The employment of the most environmentally damaging gas delivery system at MREH (the gas cylinders), currently results in a 63 times higher CO_2_ emission per RRD repair compared to UHCW.

Over the four years an average of 688 primary RRD and 82 re-detachment RRDs (770 RRDs in total) were repaired annually at MREH and BMEC combined. Based on the 2011 census, Greater Birmingham and Greater Manchester had a combined population of 4,994,365 [13]. Based on our data, this gives an annual incidence of 13.78 RRD repair per 100,000 people. Extrapolated to the entire UK population (63,02 million according to the 2011 census) we estimate that 8,707 primary RRD repairs are performed annually in the UK. Assuming that 30% of all RRDs are suitable to be repaired with AT as it was the case at UHCW, this could lead to 2,921 fewer RRDs repaired with fluorinated gas annually across the UK. This would correspond to a 44.3% to 56.6% reduction in CO_2_ emissions, depending on the gas source used (30ml single-use gas cannisters versus cylinders). Based on our mean CO_2_ equivalent emissions per patient, the use of AT could result in a reduction of up to 716.5 tons of CO_2_ nationally every year which corresponds to savings in electricity for up to 121 homes annually [10].

## Discussion

In this study we found that using AT in 30% of RRD repairs could reduce CO_2_ emissions by half. To the authors’ best knowledge, this is the first study to quantify the CO_2_ emissions due to gas tamponade in RRD surgery and the effect of introducing the use of AT in suitable cases.

The longevity and absorption of fluorinated gases in the eye do vary depending on the type of gas [14]. Long-acting gas tamponades provide longer retinal support compared to AT which is particularly important for inferior retinal detachments, large breaks and multiple breaks. Longer-acting fluorinated gases, however, have several disadvantages compared to AT including prolonged post-operative visual disruption, air travel restrictions, other high-altitudinal activities and increased risk of human operational error when calculating gas concentrations [15]. AT will result in a reduction in intraocular pressure fluctuations [16] and can therefore reduce the need for day one review post vitrectomy. This can further contribute to the overall reduction in emissions. Despite these advantages, a national study conducted in the UK showed that AT accounted for just 1.0% of all RRD repairs [17].

AT has been repeatedly demonstrated to have an excellent safety profile in the literature [6,14,16,18–27] and Moussa et al. demonstrated an excellent safety profile of AT in the management of RRD by DYP at UHCW in a previously published prospective case series [6]. In this case series, AT was performed on patients that fulfilled the Pneumatic Retinopexy vs. Vitrectomy for Retinal Detachment Trial (PIVOT) inclusion criteria of: i) A single retinal break or group of breaks, no larger than one clock hour in detached retina, ii) all breaks in detached retina to lie above the 8 and 4 o’clock meridian and iii) breaks or lattice degeneration in attached retina at any location (even inferior) were allowed [5]. With this criteria, patients had a 96% primary attachment rate with three months follow-up. Only one case series by Tan et al noted that gas tamponade was superior than AT for inferior retinal detachments, but this difference was not evident with superior RRD [18]. Several comparative case series and an randomised control trial (RCT) have published no difference in primary re-detachment rate between air and gas tamponade [16,23,28]. Even in the case of inferior RRD, three case series and one RCT demonstrate comparable safety profile using AT vs. gas tamponade [16,23–25]. Using this criteria, UHCW managed to repair 30% of RRD with AT, and the best of the authors knowledge, UHCW remains the only centre in the UK routinely using AT for RRD repair. There are several papers that estimate the proportion of retinal detachments that fulfil the PIVOT trial criteria (and therefore be eligible for AT as per the inclusion and exclusion criteria in this manuscript) [6,29], and these range from 27% to 52.9%. Therefore, compared to the largest case series in the literature, that estimates 52.9% over 1,091 patients fulfilled the PIVOT trial criteria [29], our 30% is a conservative estimate.

The more patients that can be safely operated on with AT, the larger the reduction in CO_2_ emissions, but this must be done with patient safety and surgeon preference in mind.

As SF_6_ is both, the most environmentally damaging and shortest acting fluorinated gas, this makes it the most conceivable tamponade choice to replace with air. Given that even inferior RRDs have had excellent outcomes with AT, and most clinicians do not utilise SF_6_ for inferior RRDs, the switch to AT is worth considering. Pak et al. reported a 94.4% success in primary RRD repair in 71 patients with exclusive use of AT, and no predetermined treatment criteria [28]. Although this criterion is unlikely to be widely attributable across a variety of patient populations and surgeons.

As shown with our data, the included centres have widely different tamponade choice for RRD repair (Table 2). MREH in particular, utilise SF_6_ in over half of all their RRD repairs. Even considering the effect of different populations, this implies that there are multiple effective ways to treat an RRD and different tolerances between clinicians to what constitutes a ‘safe RRD’ for SF_6_ tamponade. As such, defined agreed departmental protocols for using AT may improve consistency between clinicians, and increase its use.

If a longer tamponade duration is required than AT, but not to the typical duration that C_2_F_6_ lasts, a lower concentration of C_2_F_6_ could be considered as a replacement to SF_6_.

Although Ophthalmology accounts for a small portion of CO_2_ emissions, changes of various degree across multiple disciplines of medicine will total to significant reductions in our NHS carbon footprint and it is reassuring to see many efforts in this direction [30–32]. Although there are significant environmental savings that can achieved using AT, the net savings compared to other industries are small, and patients’ safety and surgeon preference (and confidence in their surgical technique) must take precedence.

### Study limitations and strengths

This is a retrospective study without an agreed protocol for the choice of gas tamponade to be used according to case complexity. The study strengths, however, lie in the large number of cases collected over several years, reflecting the real-life fluorinated gas use in RRD repair at large VR centres in the UK. The CO_2_ equivalent figures calculated using three different gas delivery methods should be applicable to VR centres around the world.

Although this study primarily investigates AT in RRD repair, the effect of the gas delivery system also has a profound impact on CO_2_ emissions, and it should be further investigated in future studies. The reduction of CO_2_ emissions by using AT and efficient gas delivery methods are only two aspects to make VR surgery more environmentally friendly. Other factors such as intraocular gas choice, anaesthetic technique, theatre time utilisation, equipment use and follow up protocols will contribute to the overall carbon footprint of a patient undergoing a VR operation. Further studies are warranted to assess the entire pathway.

## Conclusions

We present the real-life use and the environmental effects of fluorinated gases and AT in the management of RRDs, and highlight the potential benefits of the use of AT in selected cases in reducing the carbon footprint of the VR surgery. Further studies are required to determine the most ‘environment-friendly’ intraocular tamponade without compromising patient outcomes.

## Supporting information

S1 File Raw data. (XLSX)
